# Unusual Presentation of Acute Annular Urticaria: A Case Report

**DOI:** 10.1155/2011/604390

**Published:** 2011-09-11

**Authors:** Gilles Guerrier, Jean-Marc Daronat, Roger Deltour

**Affiliations:** ^1^Epicentre, 75011 Paris, France; ^2^Agence de Santé, Mata Utu, 986 Wallis, France

## Abstract

Acute urticarial lesions may display central clearing with ecchymotic or haemorrhagic hue, often misdiagnosed as erythema multiforme, serum-sickness-like reactions, or urticarial vasculitis. We report a case of acute annular urticaria with unusual presentation occurring in a 20-month-old child to emphasize the distinctive morphologic manifestations in a single disease. Clinicians who care for children should be able to differentiate acute urticaria from its clinical mimics. A directed history and physical examination can reliably orientate necessary diagnostic testing and allow for appropriate treatment.

## 1. Introduction

Acute annular urticaria, an acute urticarial hypersensitivity syndrome, is a morphologic subtype of urticaria characterized by the acute onset of blanchable annular, arcuate, and polycyclic erythematous wheals that subsequently fade within hours. However, lesions may display central clearing with ecchymotic or haemorrhagic hue, often misdiagnosed as erythema multiforme, serum-sickness-like reactions, or urticarial vasculitis (Table 1). We report a case of acute annular urticaria with unusual presentation occurring in a 20-month-old child to emphasize the distinctive morphologic manifestations in a single disease. 

## 2. Case Presentation

**Figure 1:**
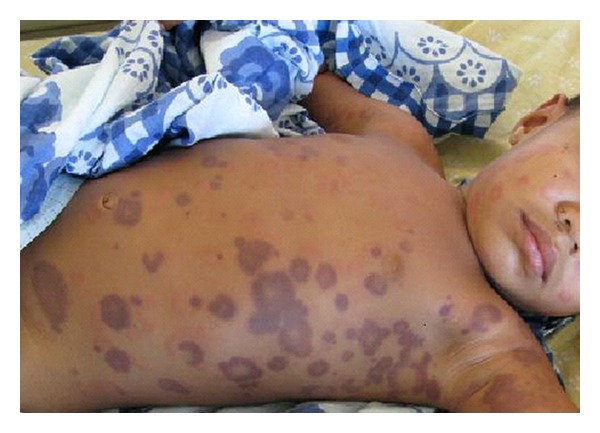
Annular, arcuate, and polycyclic wheals with central clearing and purpuric hue on the trunk.

**Figure 2:**
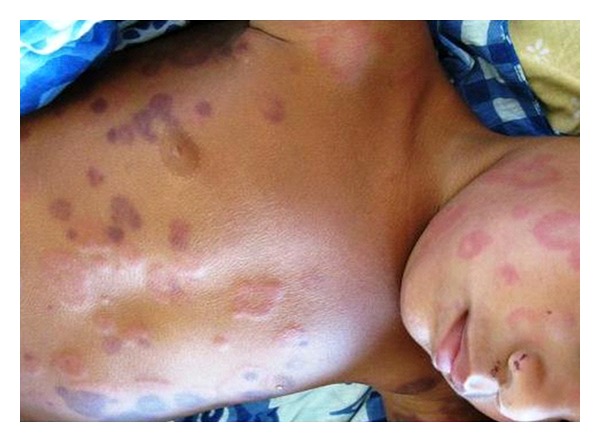
Second eruption featuring small urticarial papules on face, trunk, and limbs.

A 20-month-old boy was hospitalised in our ward with the presence of erythematous-purpuric lesions, roundish in shape, not itchy, 1 to 5 cm in diameter, localized on the trunk, the neck, and upper limbs (Figure 1). His history revealed that the eruption developed suddenly two hours ago, while an upper respiratory tract infection with mild fever was treated with aspirin during the previous days. The overall general condition was good. There was no dermographism. Mucous membranes and joints were not involved, and there was no peripheral oedema. The haematological parameters were within normal limits. On admission, the patient was treated with amoxicillin and systemic antihistamines. A second urticarial eruption without any haemorrhagic hue appeared the next day on the face, the trunk, and limbs (Figure 2). This rash faded few hours once amoxicillin was replaced by macrolides. One week after admission, the individual lesions from the first eruption resolved without any sequelae. Levels of C3 and C4 were within normal limits, as were IgA and IgE levels.

## 3. Discussion

Acute annular urticaria is reportedly more common in children 4 months to 4 years of age [1]. The diagnosis is typically made on clinical grounds and should not require skin biopsy. Although absent in our case, facial and/or acral edema is commonly seen in urticaria, with a reported prevalence of 60% to 89% [2, 3]. Pruritus, an almost universal finding associated with urticaria, has been reported in up to 94% of cases [4]. Many children with urticaria have a history of infection or recent use of a systemic medication, often an antibiotic or an antipyretic. Viral infection is the more frequently associated pathogen at all ages [5]. In our case, the second eruption might have possibly been avoided, would amoxicillin not been prescribed, considering viral infection as the most likely cause for the first eruption (normal haematological examination, low fever, and upper respiratory tract infection). Moreover, choice of antibiotic was not in line with recent guidelines for the diagnosis of allergy to antibiotics or additives [6]. Aspirin may have played a role in worsening the first eruption, which lasted longer than usually observed in acute urticaria. Although antihistamines were discutable while itching was not present in our case, the majority of patients require combinations of systemic antihistamines to achieve satisfactory symptomatic relief. Systemic corticosteroids are required only in patients remaining symptomatic despite combination of antihistamine therapy.

Acute annular urticaria is underrecognized as a result of the paucity of reported cases in the literature mainly due to similarities between distinct clinical entities, such as erythema multiform or serum-sickness-like reactions. It has been our experience that erythema multiforme is incorrectly diagnosed in many patients with an urticarial drug eruption because the edema from the urticaria can result in some surrounding pallor and some central pallor or duskiness, which can mimic a type of targetoid pattern. An important distinction is the fleeting duration of the lesions of urticaria, which usually last minutes to hours as opposed to the fixed lesions of erythema multiform and serum-sickness-like reactions, which typically last from days to weeks. Erythema multiform represents a self-limiting cutaneous cytotoxic hypersensitivity reaction mostly due to Herpes simplex virus and generally requires symptomatic treatment only.

Serum sickness-like reactions are a recognizable reaction to a drug. It has occurred most frequently following cefaclor, but may occur with other cephalosporins, penicillins, or other drugs. Prior treatment with the drug is not necessary. The acute onset in a child of inflammatory, red papulonodules that spread out into annular, urticarial plaques with dusky centers is characteristic. The onset is usually 7–10 days after the causative drug was begun. There are no true target lesions as with erythema multiforme, and the lesions may expand but do not have the day-to-day fluctuation seen in urticaria. The child often has joint pains and fever. Lymphadenopathy and renal involvement usually do not occur, in contrast to true serum sickness. The causative medication should be stopped. Corticosteroids can be dramatically helpful, if needed. 

Eruption in urticarial vasculitis is characterised by hives with a dusky purpuric centre and usually resolves with postinflammatory dyspigmentation. Although drugs are sometimes implicated in urticarial vasculitis, the condition is most often idiopathic; other causes include infections, autoimmune processes, and neoplastic processes. When distinguishing between urticarial vasculitis and urticarial drug eruption on the basis of clinical clues proves difficult, a complete blood cell count, sedimentation rate, urinalysis, and skin biopsy can be helpful. The treatment of this condition includes discontinuation of the offending agent, administration of systemic antihistamines, and administration of a 2- to 3-week course of systemic corticosteroids for more severe symptomatic cases.

Clinicians who care for children should be able to differentiate acute urticaria from its clinical mimics. A directed history and physical examination can reliably orientate necessary diagnostic testing and allow for appropriate treatment. 

## Figures and Tables

**Table 1:** Distinguishing features of annular urticaria, erythema multiforme, and serum-sickness-like reactions.

Feature	Annular urticaria	Erythema multiforme	Serum-sickness-like reactions	Urticarial vasculitis
Appearance of individual lesions	Annular and polycyclic wheals with central clearing or ecchymotic centres	Classic target lesion with purpuric or dulsy, violaceous centre (may blister), middle ring of pallor and edema, outer ring of erythema or blisters	Polycyclic urticarial wheal with central clearing, may appear purpuric	Hives with dusky purpuric centre

Location	Trunk, extremities, face	Involvement of palms, soles common	Trunk, extremities, face, lateral borders of hands and feet	Trunk, extremities, face, lateral borders of hands and feet

Duration of individual lesions	<24 h	Days to weeks	Days to weeks	Days to weeks

Fixed lesions	No	Yes	Yes	Yes

Total duration of rash	2–12 days	2-3 weeks	1–6 weeks	1–6 weeks

Mucous membrane involvement	Oral edema common, no erosions or blisters	May see oral erosions or blisters of lips, buccal mucosa, and tongue, rarely involves conjunctiva, nasal, or urogenital mucosa, usually involving only a single site	Oral edema common, no erosions or blisters	May see oral erosions or blisters of lips, buccal mucosa, and tongue, may involve conjunctiva, nasal, or urogenital mucosa, may involve several sites

Facial or acral edema	Common	Rare	Common	Common

Dermographism	Yes	No	No	No

Fever	Occasionally, low grade	Occasionally, low grade	Prominent, high grade	Variable

Associated symptoms	Pruritus	Mild pruritus or burning	Myalgias, arthralgias, lymphadenopathy	Variable

Common triggers	Viral illness, antibiotics, immunizations	Herpes simplex virus, other viral illness	Antibiotics	Infections, autoimmune processes, neoplastic processes, drugs

Treatment	Discontinue any new or unnecessary antibiotics or medications, combinations of H1 and H2 antihistamines may be helpful, systemic steroids can be helpful in more recalcitrant cases	Supportive care, early institution of systemic steroids can sometimes be helpful	Discontinue any new antibiotics or medications, H1 and H2 antihistamines, supportive care, consider systemic steroids	Discontinue any new antibiotics or medications, H1 and H2 antihistamines, supportive care, consider systemic steroids
